# Review of the Clinical Approaches to the Use of Urine-based Tumor Markers in Bladder Cancer

**DOI:** 10.5041/RMMJ.10314

**Published:** 2017-10-16

**Authors:** Timothy Clinton, Yair Lotan

**Affiliations:** Department of Urology, UT Southwestern Medical Center, Dallas, Texas, USA

**Keywords:** Bladder cancer, screening, surveillance, urine tumor markers

## Abstract

Bladder cancer is a common disease with a stable incidence for the past few decades despite advancements in molecular and genetic determinants of cancer development and progression. Cystoscopy remains the standard for detection and surveillance of bladder cancer, but it is an invasive and potentially costly procedure. With the knowledge of molecular alterations associated with bladder cancer numerous urine-based tumor markers have become commercially available. These urine markers have been evaluated in all clinical scenarios for the detection of bladder cancer including screening, hematuria, atypical cytology evaluation, and surveillance, but given the relative lack of impactful trials they are not routinely utilized. The efforts to develop markers with increased sensitivity to replace cystoscopy for the detection of bladder cancer have thus far been unsuccessful as well. This review addresses role of urine markers for screening, detection, and surveillance of bladder cancer.

## INTRODUCTION

Bladder cancer remains the fifth most frequent non-cutaneous malignancy in the United States with over 79,000 estimated new cases for 2017.^1^ The incidence of bladder cancer has remained stable from 1988 to 2006, and similarly the distribution of bladder cancer by stage has remained stable from 2004 to 2010 based on evaluation of the Surveillance, Epidemiology and End Results (SEER) database.^2^,^3^ Despite advancements in biologic processes, genetics, diagnosis, treatment, and surveillance of bladder cancer the role of markers in identifying disease is still unclear. Urothelial carcinoma is by far the most common histologic subtype. The risk factors for urothelial carcinoma have been well established, with the most common being cigarette smoking.^4^ Approximately 75% of newly diagnosed bladder cancer cases present as non-muscle invasive bladder cancer (NMIBC).^5^ With these non-invasive tumors the main treatment is bladder-sparing surgery with transurethral resection of tumor as well as a risk-based stratification of use of intravesical chemotherapy or immunotherapy to decrease risk of recurrence and/or progression.^6^,^7^ Unfortunately, despite attempts at initial control of NMIBC, 50%–70% recur, with 10%–20% progressing to muscle-invasive bladder cancer (MIBC).^5^ Given the high relapse rates of NMIBC and the possibility of progression, frequent patient monitoring and surveillance is required.

The advances in understanding bladder cancer at the molecular and genetic level have led to the identification of detectable and measurable alterations associated with the disease. Given the ease of obtaining voided urine and its direct contact with potentially malignant urothelium, numerous urine-based tumor marker tests have been generated. While white-light cystoscopy remains the standard for detection of bladder cancer, it is recognized that enhanced cystoscopy with blue light or narrow-band imaging frequently detects tumors that are missed by white light.^8^,^9^ In fact the American Urological Association (AUA) guidelines for managing NMIBC state that “in a patient with NMIBC, a clinician should offer blue light cystoscopy at the time of TURBT [transurethral resection of bladder tumor], if available, to increase detection and decrease recurrence (Moderate Recommendation; Grade B).”^7^

While enhanced cystoscopy is currently available in the operating room, it is still not readily accessible in clinic for office cystoscopy. Hence urine markers have been investigated to replace or augment the accuracy of cystoscopy in the office setting primarily. At the time of writing, seven tests are comercially available: six are approved by the US Food and Drug Administration (FDA), and one meets Clinical Laboratory Improvement Act standards (Table 1).^10^ Currently NMP22, NMP22 BladderChek, and UroVysion all have FDA approval for detection of bladder cancer and in surveillance, while uCyt+, BTA TRAK, and BTA STAT only have approval for surveillance.^10^ This review will approach each step of current clinical approaches from diagnosis to surveillance of bladder cancer and what role urine markers can play in medical decision-making.

**Table 1:** Commercially Available Urine-based Tumor Markers.

Marker Type	Test	Manufacturer
Protein-based	NMP22 NMP22 BladderChek	Alere, Waltham, MA, USA
	BTA TRAK BTA STAT	Polymedco, Cortlandt Manor, NY, USA
Cytologic immunohistochemistry	uCyt+ (Immunocyt)	Scimedx Inc., Denville, NJ, USA
Genetic-based	UroVysion FISH	Abbott Inc., Abbott Park, IL, USA
	Cxbladder	Pacific Edge Inc., Dunedin, New Zealand

## DEVELOPMENT OF A CLINICALLY USEFUL MARKER

A number of tests are available for screening, diagnosis, and staging of bladder cancer. The requirement for a biomarker to be valuable is that it needs to provide a benefit or improvement over the current standard evaluation. As has previously been described, an ideal biomarker needs to be “easier, better, faster, cheaper” than current standards.^11^ Point-of-care tests like NMP22 BladderChek and BTA STAT can provide information during an office visit that may allow more timely decision-making. However, the more sophisticated markers often require more time to perform. Cost is also an issue for markers that rely on more technical expertise.

While a majority of studies have focused on the detection and development of markers, there is a lack of quality assessment in prospective clinical trials. This review will focus on the following clinical scenarios:

- Screening
- Hematuria evaluation
- Atypical cytology or cystoscopic findings
- Surveillance of bladder cancer

## SCREENING POPULATION

Bladder cancer mortality has remained relatively stable over the last few decades despite improvements in surveillance and treatment.^12^ Cancer screening has been shown to cause a major reduction in cancer mortality in breast, cervical, and colon cancer.^13^ Bladder cancer is a favorable candidate for screening, given the ease of collection of urine and its direct contact with potentially malignant urothelium, but at this time the US Preventive Services Task Force has deemed that there is insufficient evidence to support screening for bladder cancer.^14^ Unfortunately, early detection is vital in bladder cancer, given the stage-dependent survival rates and the fact that 25% of patients present with invasive or metastatic disease.^12^ Therefore, if screening can identify high-grade bladder cancer at an earlier asymptomatic stage this could translate to improved survival. Identifying a population with sufficient incidence is critical.^15^,^16^

### Prior Screening Studies

Initial screening methods began with simple hemoglobin urine dipstick testing at home. Messing et al. prospectively screened 1575 men with 14 days of hemoglobin dipstick testing and found 258 (16%) with positive chemical dipstick results for hematuria, with 21 (1.3%) diagnosed with bladder cancer.^17^,^18^ The long-term follow-up study found that 4.8% of screen-detected tumors were muscle-invasive compared to 23.6% of a non-screened population, thereby demonstrating downstaging of bladder cancer due to screening.^18^ In the 14 years of follow-up, bladder cancer deaths were seen in 20% of unscreened patients but in none in the screened population. The weakness of this study was the lack of a randomized control arm. Other studies evaluating urine dipstick have had some conflicting results. A study by Britton et al. screened 2356 men with urine dipstick; 474 (20%) had hematuria, of whom 319 underwent evaluation.^19^ This diagnosed only 17 asymptomatic NMIBC, but long-term follow-up demonstrated 5 patients who progressed and 3 who died of the disease. This study has many weaknesses, including likely understaging at the time of diagnosis.

While the use of hemoglobin dipstick is a cheap and manageable option for widespread screening, there are multiple urine-based tests that have superior sensitivities. Hence secondary screening with a urine marker may reduce number of cystoscopies needed. Roobol et al. evaluated a Dutch population with urine dipstick, and those subjects with microscopic hematuria underwent further urine molecular tests with NMP22, FGFR3, microsatellite, and methylation analysis; those who were positive underwent cystoscopic evaluation.^20^,^21^ Of the 1747 men who completed the protocol 409 (23.4%) had hematuria, of whom 75 had at least one positive urine marker test, with only 4 non-invasive bladder tumors and 1 kidney cancer identified.

Given the limitations of prior studies due to the low prevalence of bladder cancer in screened populations, investigators began evaluating the utility of screening in high-risk populations. The risk factors associated with bladder cancer have been well delineated, and this led most investigators to focus on those with a smoking history or an occupational carcinogen exposure.^16^,^22^ Steiner et al. screened 183 patients with a greater than 40 pack-year smoking history with urine dipstick, NMP22, cytology, and UroVysion and identified 75 patients with at least one positive test.^22^ These patients underwent cystoscopic evaluation, and 3 were found to have NMIBC and 12 had either dysplasia or inverted papilloma. A larger study from Lotan et al. also screened a high-risk population with age over 50 and greater than 10-year smoking history or a significant high-risk occupation such as working with dyes, petroleum, or the chemical industry.^16^ In total 1502 patients were screened with NMP22 BladderChek test, 85 (5.7%) having a positive test. Of these, 69 underwent cystoscopic evaluation, with identification of 2 NMIBC (one high-grade and one low-grade) and 1 patient with marked atypia.

### Potential Role of Markers for Screening

The National Cancer Institute lists two requirements that must be met for screening protocols to be efficacious: (1) the disease can be detected earlier than if the disease symptoms appear, and (2) there is evidence that treatment initiated earlier due to screening can result in an improved outcome.^23^–^25^ Identifying a high-risk population will be necessary for screening to be justified. Multiple studies have created individualized risk scores and predictive models that may predict the subgroups that are most at risk.^26^,^27^ One study has utilized a cost-effectiveness model to identify a screening population and concluded that the ideal population would be one that has an incidence of bladder cancer of greater than 1.6%.^23^

## HEMATURIA EVALUATION

Hematuria (gross or microscopic) is the main symptom that leads to bladder cancer diagnosis, with an incidence of about 10% in those with gross hematuria and 2%–5% in those with microscopic hematuria.^28^ The American Urological Association (AUA) in 2012 developed guidelines for asymptomatic microscopic hematuria (AMH), defined as 3 red blood cells or greater per high-power field, and recommend cystoscopy for all adults over 35 with microscopic hematuria.^29^ Unfortunately AMH is also seen in 9%–18% of normal individuals; thus practitioners are faced with the decision of who should undergo this complete evaluation. Studies have now shown that a majority of patients with AMH fail to get referred to a urologist for cystoscopic evaluation.^30^,^31^ Delayed referral can result in a more advanced stage and hence a worse prognosis.^32^ Therefore, improving evaluation of patients with gross and microscopic hematuria may lead to more timely referral and earlier detection of the disease. Urine markers as well as clinical factors may play a critical role in risk-stratifying patients and possibly reducing the need to perform cystoscopy on very low-risk patients.

### Current Standard Hematuria Evaluation

Hematuria evaluation currently utilizes cystoscopy for direct endoscopic evaluation of the bladder and imaging for evaluation of upper tract or renal abnormalities.^29^ The use of cystoscopy is the standard in bladder cancer diagnosis, with a high sensitivity, but false negative rates are variable and can be over 10%.^29^,^33^ Therefore, practitioners will often utilize cytology as an adjunct test to aid in the detection of bladder cancer. Cytology is not recommended for every case by the AUA AMH guidelines but rather is only suggested as an option in those with persistent AMH after a negative evaluation or in those with risk factors for carcinoma *in situ* (CIS), such as irritative voiding symptoms, tobacco use, and chemical exposures.^29^ Cytology remains the adjunct test of choice due to its overall high specificity of 95%–99%.^34^ There are many limitations to the use of cytology, including its low sensitivity especially for low-grade cancer, interobserver variability, and atypical findings. Even though urine marker tests provide valuable information and almost all have a higher sensitivity than cytology for low-grade cancer, they are not routinely used due to the high rate of false positives leading to unnecessary and costly diagnostic procedures and increased patient anxiety.^34^

### Potential Role of Markers in Hematuria Evaluation

Given the low referral rates to urologists for hematuria evaluation, markers can potentially identify or stratify which patients truly need an evaluation. Lotan et al. developed a bladder cancer nomogram utilizing age, gender, smoking status, ethnicity, hematuria, and NMP22 BladderChek to define those most at risk of bladder cancer at time of cystoscopy.^35^ The initial evaluation of the nomogram of 1272 patients had a predictive accuracy of 0.82. This model was then validated in a prospective multicenter study for those patients referred for hematuria evaluation.^36^ Of 381 patients, 23 (6%) were found to have bladder cancer, and the predictive accuracy remained similar at 0.79. The main role of risk stratification would be to triage high-risk patients earlier and avoid cystoscopy in low-risk patients, such as women who do not smoke and have a negative marker. The safety of such approaches need validation.

The NMP22 BladderChek test evaluates a single protein in the urine, but there are recently developed panels of markers which may have improved sensitivity (Table 2). One such test is a new RNA assay, Cxbladder Detect, which has yet to be approved by the FDA but meets Clinical Improvement Act standards. In a prospective trial of 485 patients presenting with gross hematuria the bladder cancer incidence was 66 (13.6%), and the test performed with a sensitivity of 81.8% and specificity of 85.1%.^40^ Further stratification with removal of low-grade tumors increased the sensitivity and specificity to 91% and 90%, respectively. Using the same RNA assay, Cxbladder Triage is a model that combines phenotypic factors (age, gender, frequency of macrohematuria, and smoking history) with genotypic variables in a voided urine sample.^41^ This model helps to triage patients presenting with hematuria from having to undergo a complete evaluation. The findings in the small discovery study of gross hematuria found a sensitivity of 95% and negative predictive value of 97%. Further analysis of physician clinical decisions demonstrated that the Cxbladder test improves the management of asymptomatic hematuria by reducing the number of invasive procedures.^42^ If validated, Cxbladder Detect and Triage may replace cytology not only as an adjunct test but may possibly replace cystoscopy in certain settings.

**Table 2:** Performance of Markers in Surveillance Setting from Pooled Analyses (Adapted from Chou et al.^37^).

Test/Marker	Sensitivity (95% CI)	Specificity (95% CI)
Cytology^38^	0.35 (0.13–0.75)	0.94 (0.85–1.00)
NMP22	0.61 (0.49–0.71)	0.84 (0.75–0.90)
NMP22 BladderChek	0.70 (0.40–0.89)	0.83 (0.75–0.89)
BTA TRAK	0.58 (0.46–0.69)	0.79 (0.72–0.85)
BTA STAT	0.60 (0.55–0.65)	0.76 (0.69–0.83)
uCyt+	0.75 (0.64–0.83)	0.76 (0.70–0.81)
UroVysion FISH	0.55 (0.36–0.72)	0.80 (0.66–0.89)
Cxbladder Monitor^39^	0.93	NA

CI, confidence interval.

## ATYPICAL CYTOLOGY

### Potential Role of Markers in Evaluation of Atypical Cytology

One of the main limitations of cytology is its often inconclusive findings with atypia. This cytology report creates a dilemma for urologists and patients in the setting of a negative cystoscopy, suspicious cystoscopy, or a patient with history of bladder cancer. Beyond the anxiety for patients, urologists must determine whether to observe the patient, with the risk of missed cancer resulting in progression, or biopsy every patient with atypia, placing the patient at risk of anesthesia and the procedure itself. To complicate the dilemma further, atypical cytology when biopsied can demonstrate malignancy in up to 23% of samples.^43^ Given the increased sensitivity of many urine markers such as fluorescence *in situ* hybridization (FISH) and uCyt+ and the high specificity of cytology, studies have shown that the combined analysis of these two tests reaches a high sensitivity and specificity.^44^–^46^ This has led to the evaluation of second-test reflex UroVysion and uCty+ testing in those with atypical or negative cytology.^47^–^51^ A prospective evaluation and subsequent validation study was performed by Lotan et al. and found a high sensitivity of the FISH assay to detect bladder cancer in patients with atypical cytology and an equivocal or negative cystoscopy.^49^,^50^ UroVysion had a high negative predictive value (NPV) and reasonable positive predictive value (PPV), such that patients with a negative UroVysion could avoid further evaluation and those with positive testing could be monitored more carefully or biopsied. Given these studies, urine marker testing for equivocal cytology has been included in the AUA NMIBC guidelines for those previously diagnosed with bladder cancer.^7^

## SURVEILLANCE OF BLADDER CANCER

Surveillance of patients with bladder cancer has been the main focus for the use of diagnostic markers. In surveillance settings a test with maximal sensitivity is of utmost importance, and most of the new urinary marker tests have increased overall sensitivity as compared to cytology, but this is mostly driven by low-grade disease (Table 2).^10^ The predictive values of tests are also greatly improved due to a higher prevalence of the disease in surveillance as compared to screening populations. The AUA NMIBC guidelines currently do not recommend the use of urinary biomarkers in place of cystoscopic evaluation.^7^

### Low-risk NMIBC Surveillance

In low-risk NMIBC, the risk of progression is low, thus delays in identifying a recurrence is unlikely to be clinically significant. Therefore, the surveillance approach of low-risk NMIBC is to limit invasive procedures. Biomarkers can be utilized in this approach to aid in the reduction of cystoscopy frequency or define situations where a cystoscopy can be safely omitted.

The sensitivity of cytology is low for low-risk bladder cancer; therefore, surveillance relies almost solely on cystoscopy. All currently approved biomarkers including cytology have lower sensitivities for recurrent disease since tumor volumes tend to be smaller.^38^ Studies have combined biomarkers in a panel, and while the sensitivity for even low-risk bladder cancer was increased to as high as 93% the specificity was dismally low.^52^ Individual markers have been examined to determine whether they can replace cystoscopy, but none achieve a sensitivity sufficient to replace it.^53^,^54^

The use of biomarkers in low-risk bladder cancer will have to prove to be cost-effective, especially in a disease with minimal risk of progression such that some even consider watchful waiting.^55^,^56^ The use of biomarkers should therefore provide a surveillance protocol that can reduce the frequency of cystoscopy. An example of a modified surveillance approach has been demonstrated to be cost-effective while also decreasing the frequency of cystoscopy.^57^ The study investigated the sensitivity and cost of a range of urine markers and cystoscopy, and identified a cost-effective regimen of urine marker tests and cystoscopy alternating every 3 months. The model of cost-effective modified surveillance protocols requires prospective evaluation in a randomized trial to assess not only progression-free and overall survival but the financial outcomes as well.

### Intermediate-risk NMIBC Surveillance

The intermediate-risk bladder cancer classification has historically been a catch-all for tumors that did not satisfy either low-risk or high-risk.^58^ Recently further identification of risk factors, such as multiple low-grade tumors, low-grade tumor size ≥3 cm, early recurrence <1 year, or frequent recurrences, has provided evidence that some low-grade tumors harbor a more aggressive biology than previously thought.^59^ Therefore modified surveillance and treatment protocols for intravesical therapy have been developed specifically for this intermediate-risk group.^7^,^58^,^60^ Incorporating markers may allow more stringent monitoring without increasing cystoscopy procedures by alternating markers and cystoscopy. For example, a test such as Cxbladder Monitor which is designed to maximize sensitivity may be able to serve as a rule-out test so only patients with a positive test will undergo cystoscopy.^39^ Other benefits were seen in a study that found that urologists who knew a patient had a positive test prior to cystoscopy were more likely to find a cancer.^61^ These different uses of biomarkers for augmented cystoscopic detection are ideal for these low-risk and intermediate-risk tumors as more urologists are able readily to treat these recurrent tumors with office-based fulguration, thereby limiting any delay to treatment or anesthetic risks.

### High-risk NMIBC Surveillance

High-risk NMIBC is of great concern in surveillance due to its high risk of recurrence and progression. The sensitivity of markers alone is not sufficiently high to replace cystoscopy, but they have shown that adjunct use is beneficial. The NMP22 BladderChek test has been shown to increase the sensitivity of cystoscopy from 91.3% to 99.0% in those undergoing surveillance, for example.^62^ Recent evaluation of Cxbladder Monitor in those patients undergoing surveillance for NMIBC has demonstrated superiority with sensitivity of 91% and NPV of 96% as compared to current urine markers including cytology^63^ There is still a need for studies that demonstrate cost-effectiveness and improved clinical management of high-grade cancer.

When developing a marker, it is important to identify the clinical significance of a positive result. For example, there are very few false positive results with cytology, and therefore the clinical management can rely on these findings. Unfortunately, there is insufficient evidence to determine what to do with a positive marker in the setting of a normal cystoscopy. Since specificity of common markers is no better than 75%, many positive results can either be false or anticipatory. An anticipatory result means that the test is detecting microscopic disease, which may recur earlier but is not currently detectable. There is no clear intervention that can be used in this setting, and most of high-risk patients are already on maintenance Bacillus Calmette–Guérin therapy (BCG).

For example, patients with a positive UroVysion FISH test have a shorter time to recurrence than those whose test was negative, but the average time to recurrence was 12 months so it is not clear how management should change.^48^,^64^ It is still unclear how to incorporate markers in surveillance of high-risk disease, but a highly sensitive and specific marker could replace cytology.

### Assess Response to BCG

Intravesical immunotherapy treatment with BCG is the standard of care in patients with high-risk bladder cancer and in some with intermediate risk.^7^,^58^ While BCG has been shown to decrease tumor recurrence and progression, there have been no studies to identify which tumors will fail treatment, i.e. those that are BCG-refractory. UroVysion is a potential test for monitoring failure of intravesical BCG treatment.^65^ In fact a recent prospective trial showed that UroVysion could predict response to BCG.^66^ Given the utility of this test, the AUA guidelines have included the use of UroVysion to assess response to BCG.^7^ This use of biomarkers to identify those that may benefit from certain treatments is the first step to developing individualized treatment for patients. These findings still need validation.

## FUTURE DEVELOPMENTS

Use of urine markers has been limited due to a lack of efficient validation and effective integration into clinical decision-making. Given the vast number of studies and trials of the different markers, the differing study designs, patient selection, tumor prevalence, distribution of tumor grade and stage, different cutoff test values, and trial endpoints have made comparative analysis nearly impossible.^67^ There have been many studies and consortiums that have attempted to standardize the evaluation of molecular markers.^68^ The recent advancements of genetic information has led to the identification of new candidate molecular biomarkers. The preliminary results of some of these new biomarkers are promising, but there remains much more evaluation to identify the ideal marker that is “easier, better, faster, cheaper.”^11^ Validating the results of biomarkers remains a challenge since it is necessary to perform prospective clinical trials to demonstrate added clinical value and, in today’s financial environment, improved cost-effectiveness.^67^

## CONCLUSION

There are many potential roles for urine markers in improving the detection and monitoring of bladder cancer. Growing understanding of cancer biology is enhancing our ability to identify potential urine markers. Identification of markers is just the first step in development since validation of clinical utility is often a neglected but critical step in establishing the value of urine markers in bladder cancer care.

## Abbreviations

AMH: asymptomatic microscopic hematuria
AUA: American Urological Association
BCG: Bacillus Calmette–Guérin
CIS: carcinoma *in situ*
FDA: US Food and Drug Administration
FISH: fluorescence *in-situ* hybridization
MIBC: muscle-invasive bladder cancer
NMIBC: non-muscle invasive bladder cancer
NPV: negative predictive value
PPV: positive predictive value
SEER: Surveillance, Epidemiology and End Results (database)
TURBT: transurethral resection of bladder tumor.
